# Innovative technologies for the treatment of dry age-related macular degeneration (AMD) - modern therapeutic perspectives and their future

**DOI:** 10.22336/rjo.2025.03

**Published:** 2025

**Authors:** Przemysław Ciszewski, Alicja Drelichowska, Damian Pikor, Emilia Wiśniewska, Michał Azierski

**Affiliations:** 1 Scientific Association of Ophthalmology, Department of Ophthalmology, Silesian Medical University, Katowice, Poland; 2 Scientific Association of MedTech at the Center for Remote Learning and Educational Effects Analysis, Faculty of Medical Sciences in Katowice, Silesian Medical University, Katowice, Poland; 3 Department and Clinic of Tropical and Parasitic Diseases, University of Medical Sciences, Poznań, Poland

**Keywords:** dry age-related macular degeneration, AMD, innovative therapy, nanotechnology, AMD = Age-related Macular Degeneration, AREDS = Age-Related Eye Disease Study, AREDS2 = Age-Related Eye Disease Study 2, RPE = Retinal Pigment Epithelium, ROS = Reactive Oxygen Species, GA = Geographic Atrophy, FDA = Food and Drug Administration, SS-31 = Elamipretide (a mitochondria-targeting drug), iPSC(s) = Induced Pluripotent Stem Cell(s), ESC(s) = Embryonic Stem Cell(s), HLA = Human Leukocyte Antigen, PCL = Polycaprolactone, LLLT = Low-Level Laser Therapy, ATP = Adenosine Triphosphate, HRF = Hyperreflective Foci, OCT = Optical Coherence Tomography, FAF = Fundus Autofluorescence, AI = Artificial Intelligence, NGF = Nerve Growth Factor, RELITE = Regenerative Retinal Laser and Light Therapies, MAC = Membrane Attack Complex, sCD59 = Soluble CD59 (complement regulatory protein), PMC = Pentamethyl-6-chromanol

## Abstract

**Purpose:**

This review explores modern therapeutic options for the dry form of age-related macular degeneration (AMD), a condition representing one of the most significant challenges in ophthalmology due to its progressive nature and lack of effective treatment. The study discusses innovative approaches, evaluates available methods, and examines the potential of emerging technologies to improve patients’ quality of life.

**Methods:**

A comprehensive review of current literature was conducted, being focused on therapies for dry AMD, including classical methods such as AREDS/AREDS2 supplementation, molecularly targeted drugs, gene therapy, cell transplants, tissue engineering, nanotechnology, and light-based therapies. Emerging tools leveraging artificial intelligence for personalized treatment and predictive modeling were also evaluated.

**Results:**

AREDS/AREDS2 therapies effectively slow disease progression but cannot reverse retinal damage. Advances include molecularly targeted therapies (Pegcetacoplan, Avacincaptad Pegol) that reduce inflammation, gene therapy (HMR59) protecting RPE cells, and mitochondria-targeted drugs (SS-31) mitigating oxidative stress. Using scaffolds, nanoparticles, tissue engineering, and nanotechnology enhances RPE regeneration and drug delivery. Light-based therapies (LLLT, adaptive phototherapy) improve mitochondrial function, while AI aids in predicting disease progression and personalizing treatment.

**Conclusions:**

Modern therapeutic approaches for dry AMD provide promising avenues to slow disease progression and protect vision. However, further clinical trials are needed to optimize these strategies, assess long-term outcomes, and expand patient access to effective treatments. These advancements have the potential to significantly improve the quality of life for individuals affected by dry AMD.

## Introduction

### Characteristics of AMD

Age-related macular degeneration (AMD) is a chronic retinal disease that accounts for 7% of all cases of blindness worldwide. In addition, it is also the most common cause of blindness in developed countries in people over 60 years of age [1]. There are two main phenotypes: wet (neovascular) and dry (atrophic) forms, which differ in both pathogenesis and clinical course, as well as in the availability of treatments. The dry form of AMD is more common, accounting for approximately 80-85% of cases, but the wet form accounts for the most severe vision loss. Exudative AMD occurs in 15-20% of cases and is characterised by forming new blood vessels in the retina, leading to rapid visual degradation [2].

### Purpose of the work and methodology

This review explores modern treatment options for the dry form of age-related macular degeneration (AMD), one of the most significant challenges in ophthalmology due to the lack of effective therapies. While advances have been made in treating wet AMD, dry AMD still requires innovative approaches. The paper reviews available and emerging technologies, assessing their potential impact on patient outcomes. Literature from 2001 to 2024 was analyzed using databases like PubMed and Scopus, focusing on molecular, nanotechnological, and genetic therapies. Key studies outside this timeframe were included when highly relevant. The findings emphasize the potential of new technologies to improve clinical practices and patient quality of life.

## Dry age-related macular degeneration

### Changes in retina

The early stages of dry AMD are associated with drusen, or deposits of lipids and proteins, which accumulate due to abnormalities in the retinal pigment epithelium (RPE) and Bruch’s membrane. The size, shape, and borders of these deposits are crucial in determining the severity of the disease. Eyes with large, soft drusen and deposits that fuse are more likely to progress to more advanced stages of AMD compared to eyes with only hard drusen. Early symptoms of dry AMD also include changes in the retinal pigment epithelium, such as discoloration and atrophy, and loss of capillaries in the choroid. As the disease progresses, geographic atrophy, characteristic of advanced dry AMD, may develop. Geographic atrophy refers to a change in the retina's appearance that resembles a continent on a map, involving damage to the RPE, retina, and choroidal capillaries, with clearly defined borders of damage [3].

### Pathophysiological mechanisms

Due to its high metabolic activity, the retina is particularly susceptible to oxidative stress. Excessive production of reactive oxygen species (ROS), resulting from exposure to light and intense mitochondrial activity, leads to damage to lipids, proteins, and DNA in RPE cells. The dysfunction of these cells results in a reduced ability to degrade metabolic products, which contributes to the accumulation of druses. Their presence interferes with nutrient transport and toxin removal, leading to disease progression and gradual geographic atrophy. These changes lead to loss of macular function and deterioration of central vision [4]. Dry AMD is also associated with chronic inflammation resulting from the activation of the complement system. Oxidation products, such as lipofuscin, and cellular fragments can act as complement activation signals, further damaging retinal tissues [5].

## Classical approach in the treatment of dry AMD

### The role of AREDS and AREDS2 supplements in slowing the progression of dry AMD

The introduction of AREDS and AREDS2 supplementation represents one of the most critical advances in preventing the progression of age-related macular degeneration (AMD) [6]. The first AREDS (Age-Related Eye Disease Study) study, conducted by the National Eye Institute, showed that ARDES supplements could reduce the risk of AMD progression to late stage among patients with intermediate AMD [7,8]. This study was followed up by AREDS2, which updated the formulation of the supplements by replacing beta-carotene with lutein and zeaxanthin, which increased efficacy and minimised the risk of lung cancer progression in smokers. The results showed that lutein and zeaxanthin provided additional protection, especially in patients with low dietary intake of these components. Reducing the zinc dose to 25 mg reduced side effects without losing therapeutic efficacy [9]. Importantly, long-term analyses showed that AREDS2 supplementation could also slow the progression of the late form of dry AMD, such as geographic atrophy. The effects were particularly pronounced in areas of the retina outside the central macula (fovea), suggesting that early initiation of supplementation may delay the onset of changes in the central area responsible for central vision [10]. Studies also indicate that the response to supplementation does not depend on the individual patient’s genotype, making AREDS2 a safe and practical choice for many patients with AMD [11].

### Limitations of existing therapies

Despite their successes, AREDS and AREDS2 supplementation have limitations. Their effect is limited to slowing disease progression; it does not prevent disease progression or treat changes such as geographic atrophy. Furthermore, the inability to reverse already existing retinal damage remains a clinical challenge [10].

## Molecularly targeted therapies

### Inhibition of the complement system

The complement system is central to innate and acquired immunity, supporting immune surveillance, inflammatory responses, and homeostasis through three activation pathways: classical, alternative, and lectin. Its tasks include activating immune cells, labeling pathogens for phagocytosis and their destruction by the membrane attack complex, and tightly regulating the system to protect host cells. The dysregulation of the complement system plays a vital role in the development of AMD. Products of its activation have been found in increased amounts in patients’ plasma and deposits in ocular tissues, especially in drusen. Oxidative stress further enhances pathological processes by forming neoepitopes, which can bind to autoantibodies that activate the complement system [12].

### Pegcetacoplan and avacincaptad pegol as drugs affecting the complement system

Pegcetacoplan is a complement inhibitor that selectively targets the C3 protein, a key element in the pathophysiology of AMD. C3 is located at the top of the complement activation cascade, involving all three activation pathways, making it a desirable therapeutic target in this disease. Pegcetacoplan binds to C3 and C3b, blocking their cleavage and preventing further complement activation, thereby limiting the development of the inflammatory response and tissue damage. In February 2023, the FDA approved the intravitreal injection of Pegcetacoplan (SYFOVRE) as the first GA therapy [13]. Avacincaptad pegol is a pegylated RNA aptamer that effectively inhibits the activation of C5, a key protein in the complement cascade. Blocking C5 cleavage prevents the formation of C5a and C5b fragments, which are responsible for inflammatory reactions, cell death, and the formation of the cell membrane attack complex (C5b-9). Thus, avacincaptad pegol can slow the progression of retinal cell degeneration in GA associated with AMD, offering therapeutic potential for treating the disease [14]. The FDA approved Izervay (avacincaptad pegol) for the treatment of geographic atrophy (GA) secondary to advanced dry macular degeneration (AMD) on 4 August 2023 [15].

### Drugs targeting mitochondria

Mitochondrial dysfunction in the retinal pigment epithelium (RPE) plays a key role in the pathogenesis of dry AMD. Compounds like SS-31 (Elamipretide) exhibit antioxidant and cytoprotective effects, stabilising mitochondrial membranes and reducing oxidative stress and cellular damage. SS-31 improves retinal function in preclinical models, encouraging clinical trials to evaluate its safety and efficacy in patients with AMD. Clinical trials are underway that may provide new therapeutic perspectives for treating this disease [16].

## Gene therapy

Gene therapy for treating dry AMD relies on delivering genetic material to the eye cells, allowing for a long-term therapeutic effect without the risk of an immune system reaction [17]. HMR59, developed by Hemera Biosciences, is a gene therapy using the AAV2 vector that delivers the sCD59 protein, which is designed to protect retinal pigment epithelial (RPE) cells from damage resulting from excessive activation of the complement system in dry AMD. CD59, found on the surface of RPE cells, blocks the formation of the MAC complex, which activates a later step in the complement cascade to protect retinal tissues. Therapy involves intravitreal administration of HMR59, which leads to increased expression of CD59 on RPE cells. The treatment was well tolerated in phase 1 clinical trials, including HMR-1001. A 23% reduction in geographic atrophy (GA) and no transition to neovascularisation (nAMD) was observed at the highest dose after 18 months of follow-up. Further studies on the safety and efficacy of HMR59 in treating dry AMD are ongoing [18].

## Cellular and regenerative therapies

### RPE cell transplantation

Retinal pigment epithelial (RPE) cell transplantation is a promising strategy for treating dry macular degeneration (AMD). The primary sources of these cells are induced pluripotent stem cells (iPSCs) and embryonic stem cells (ESCs), which allow functional RPE cells to be obtained to replace degenerating retinal cells. The iPSCs are particularly attractive due to the possibility of immunological matching through iPSC banks with specific HLA haplotypes. Clinical studies indicate that iPSC-RPE transplantation is relatively safe, with minimal rejection controlled by topical steroids, and the grafts remain stable for at least one year, despite minor complications such as mild immune reactions or epiretinal membrane formation [19,20]. Long-term observations in animal models have shown that the transplantation of iPSC-derived RPE cells in rat models of retinal degeneration led to temporary preservation of visual function and photoreceptor recovery. However, progressive loss of function of transplanted cells due to fibrosis and immune responses was observed over 11 months, highlighting the need for further improvements in transplantation techniques and better control of the immune environment of the retina [21]. Clinical studies show differences in the efficacy of transplants with iPSCs and ESCs. Although ESCs are effective for retinal regeneration in dry AMD, their use is limited by ethical and immunological issues. Further research is needed to refine cell sources, differentiation methods, and transplantation techniques to ensure durable and safe clinical outcomes [19].

### Tissue engineering

The modern treatment of dry AMD uses biomaterials as scaffolds to promote RPE cell regeneration. Scaffolds provide a microenvironment that supports cell adhesion, proliferation, and differentiation, improving retinal function. Advances include 3D scaffolds with high porosity to support cell migration and nutrient transport. The thermally induced phase separation technique (TIPS) enables the production of scaffolds with controlled porosity and biodegradability that can be tailored for RPE regeneration and drug release to support AMD therapy [22]. Innovations in biomaterials also include using biodegradable polymers such as polycaprolactone (PCL) and aliphatic polymers. These materials are used in scaffolds with biomimetic properties that mimic the natural environment of RPE cells. Due to their biodegradability and metabolic conductivity, they promote tissue regeneration and integration of the scaffold into the surrounding retina. In addition, introducing nanocomposites and functionalizing scaffolds’ surfaces enhance their bioactivity and cell interactions [23]. Biomaterials and scaffolds in retinal regeneration can serve as scaffolds for cells and drug carriers, allowing controlled release of active substances to promote regeneration and inhibit retinal degeneration [24].

## Nanotechnology in the treatment of dry AMD

### Nanoparticles for drug delivery

Nanoparticles are another example of a promising future for treating dry macular degeneration. These nano-sized (109m) particles allow precise delivery of the drug and penetrate well into the tissue because of their size. In practice, nanoparticles can be divided into several subtypes, such as liposomes (spherical particles with a double phospholipid coating and hydrophilic core), nanosuspensions (hydrophobically charged nanosubstances dispersed in a colloid), nanoparticles such as nanocapsules or nanospheres (colloidal transporters composed of lipids proteins and polymer), nanomicelles (amphipathic molecules that enable lipophilic drug delivery) or dendrimers (covalently bonded molecules of therapeutic substances). One of the essential features of nanoparticles is their potential for prolonged drug release [25]. In dry macular degeneration, numerous studies have focused on nanopharmacology targeting the retinal pigment epithelium, whose cells undergo hypotrophy and degeneration in this form of the disease, resulting in visual impairment [6].

### Nanomaterials with a protective effect

As mentioned, antioxidant and anti-inflammatory substances have been shown to inhibit the pro-inflammatory effects of reactive oxygen species and nitrogen. Such substances that are commonly used in the treatment of dry AMD include the carotenoids lutein and zeaxanthin. Ge et al. (2020) developed a nanoemulsion containing, among other substances, lutein and penetratin and, based on an in situ and mouse study, demonstrated that the test nanoemulsion administered into the conjunctival sac achieved good penetration into the posterior segment of the eye, resulting in a local reduction of oxygen radicals and an improvement in retinal structure. At the same time, no toxicity of the test substance was demonstrated [26]. In contrast, Kwon et al. (2023) created a new type of cell membrane-coated nanoparticle - pegylated melanin coated with cerium oxide. Cerium is a potent antioxidant that can exist in two oxidation states, Ce3+ and Ce4+ - alternating between one state and the other allows for regeneration of the redox cycle and enables actions similar to superoxide dismutase and catalase that result in reduced free radical concentrations. Due to its content of catechol groups, melanin has strong chelating properties, enabling such a renewable change in the oxidation state of cerium. In addition, melanin itself also has antioxidant capabilities, as it captures lipofuscin, which is known for its toxic effects. Melanin molecules in the retinal pigment epithelium degenerate with age, whereas physiologically, it can synthesize or regenerate new molecules. The local introduction of its synthetic molecules could be an alternative to naturally occurring melanin [27]. Other examples of substances raising potential opportunities in the development of nano-pharmacology for dry AMD include pentamethyl-6-chromanol (PMC) from the phenol group [28] or curcumin, known for its anti-inflammatory and antioxidant properties [29].

## Light-based therapies and laser technology

### Low-energy laser therapy (LLLT)

Low-Level Laser Therapy (LLLT) is an innovative therapeutic method for treating dry macular degeneration (AMD). Its action is based on using low-energy light emitted in the red and near-infrared range, allowing retinal tissue penetration and modulation of metabolic processes in the mitochondria. A key mechanism of action of LLLT is the stimulation of cytochrome c oxidase, an enzyme involved in the electron transport chain, leading to increased adenosine triphosphate (ATP) production. Higher ATP availability promotes regeneration and protection of retinal pigment epithelial (RPE) cells while counteracting the effects of oxidative stress - one of the main factors in the pathogenesis of dry AMD [30,31]. LLLT reduces reactive oxygen species (ROS) levels in the mitochondria, crucial for protecting RPE cells. A 30% reduction in ROS was documented in a study by Zhang et al. (2024), which simultaneously indicated a 45% increase in AMPK/SIRT3 pathway activity, contributing to improved mitochondrial stability and reduced inflammation in the retina. These mechanisms also lead to improved microcirculation in the retina, increasing oxygen and nutrient availability, which promotes cell regeneration [30]. A study by Migliario et al. (2018) showed that applying 635 nm light increases ATP production by 27%, directly affecting RPE cells’ metabolic efficiency and ability to regenerate. Such effects may promote the protection of the retina against the progressive degeneration characteristic of dry AMD [31]. Clinical application of LLLT may lead to improved visual function and slower progression of dry AMD. Although visual acuity improvements of 0.2 logMAR have been reported in studies of similar type therapies, in the context of AMD, these data should be considered as potential for further verification. Glass (2021) indicated that laser therapy may benefit visual acuity and reduce markers of oxidative stress in the retina, but requires further research to confirm its efficacy in this regard unequivocally [32]. The effectiveness of LLLT depends on several parameters, such as wavelength, session duration, and energy dose. In a study by Tam et al. (2020), wavelengths of 810 nm and 635 nm were shown to be most effective in stimulating mitochondria and reducing oxidative stress, indicating the crucial importance of fine-tuning the therapy to the patient’s needs. The high efficacy of LLLT in protecting cells and improving cellular metabolism makes it a promising therapeutic option for treating dry AMD [33].

### Adaptive phototherapy

Adaptive phototherapy is an advanced therapeutic method that uses precisely controlled light to modulate metabolic processes in the retina. Unlike conventional laser therapies, adaptive phototherapy integrates the retina’s ability to adapt light to stimulate cell regeneration and improve metabolic function. This technology applies to treating macular degeneration (AMD) because it targets key pathophysiological mechanisms such as oxidative stress, mitochondrial dysfunction, and inflammation [34]. Adaptive phototherapy uses low-intensity light to reduce the production of reactive oxygen species (ROS) in the retina. In a study by Fu et al. (2020), applying 670 nm red light reduced ROS levels by 28% in laboratory models while increasing antioxidant enzyme activity in RPE cells [35]. The adaptive effects of light affect mitochondria, increasing ATP production and improving their metabolic efficiency. A study by Zueva et al. (2024) showed that controlled irradiation can increase glucose metabolism by 22%, promoting the regeneration of retinal and photoreceptor cells [36]. Adaptive phototherapy promotes neuroprotection by activating the signaling pathways responsible for retinal plasticity. A 30% increase in nerve growth factor (NGF) levels after therapy has been reported in clinical trials [37]. Adaptive phototherapy offers unique benefits for patients with AMD. In a study by Zhang et al. (2023), patients undergoing adaptive therapy experienced an improvement in visual acuity of 0.15 logMAR, corresponding to an apparent improvement in visual quality [38]. Modern systems, such as adaptive light sources with variable wavelengths, can personalize therapy depending on the severity of retinal damage and the patient’s needs. An example is RELITE (Regenerative Retinal Laser and Light Therapies) technology, which precisely integrates fluorescence diagnostics with adaptive light modulation to target damaged areas [39]. Adaptive phototherapy has excellent potential for development due to its integration with artificial intelligence and real-time retinal imaging technologies. Further research into optimizing therapy parameters, such as light intensity, exposure time, and wavelength range, could increase its effectiveness and clinical application.

## The role of artificial intelligence (AI) in personalizing dry AMD treatment

### Predictive models for GA progression

Advances in AI technology are making it possible to create accurate predictive models for assessing the risk of progression of geographic atrophy (GA) in patients with AMD. Using advanced machine learning algorithms, optical tomography (OCT) and fluorescence autofluorescence (FAF) imaging data are analyzed to identify biomarkers such as hyperreflective foci (HRF) and structural changes in the retina. A study by Schmidt-Erfurth and Bogunovic (2020) used deep learning algorithms to analyze HRFs as biomarkers of GA progression. The results showed that HRF intensity correlated with a higher risk of developing GA within 12 months [40]. Jeong et al. (2023) developed a deep-learning model that predicted GA progression based on OCT data with 89% accuracy, allowing early therapeutic intervention [41]. Incorporating AI into GA therapy trials provides a better selection of patients for clinical trials, increasing the efficiency of evaluating new therapies [42].

### Monitoring the effectiveness of treatment

Artificial intelligence-based technologies offer breakthrough tools for analyzing retinal images, enabling real-time assessment of AMD treatment efficacy. By automating image analysis, changes in retinal structure can be precisely monitored. AI assists in the analysis of OCT data, identifying subtle changes in retinal thickness and photoreceptor structure. A model developed by Lad et al. (2022) predicted changes in retinal thickness with 85% accuracy within six months of starting therapy [43]. AI-supported automated autofluorescence analysis can detect early signs of lipid degeneration in the retinal pigment epithelium (RPE), allowing for faster assessment of therapy efficacy [44]. By integrating image analysis results with predictive models, AI enables the personalization of therapy, tailoring the intensity and type of treatment to the individual patient.

## Conclusion

Dry age-related macular degeneration (AMD) represents a significant clinical challenge due to limited therapeutic options and the progressive nature of the disease leading to significant central vision loss. Modern therapeutic approaches, including molecularly targeted therapies such as complement system inhibitors (pegcetacoplan, avacincaptad pegol), gene therapies (HMR59), mitochondria-targeting drugs (SS-31), retinal pigment epithelial cell transplants, and tissue engineering, are showing promise in slowing disease progression and protecting retinal function. In addition, using nanotechnology and biomaterials to promote tissue regeneration creates new opportunities in treating dry AMD. While progress in this field has been significant, further clinical trials and precise development of therapeutic strategies are still needed to understand the long-term effects of these interventions, improve their efficacy, and provide patients with access to adequate and safe therapies that could significantly impact their quality of life.
